# Metamorphic thyroid autoimmunity in Down Syndrome: from Hashimoto’s thyroiditis to Graves’ disease and beyond

**DOI:** 10.1186/s13052-015-0197-4

**Published:** 2015-11-11

**Authors:** Tommaso Aversa, Mariella Valenzise, Mariacarolina Salerno, Andrea Corrias, Lorenzo Iughetti, Giorgio Radetti, Filippo De Luca, Malgorzata Wasniewska

**Affiliations:** Department of Pediatric, Gynecological, Microbiological and Biomedical Sciences, University of Messina, Via Consolare Valeria, 98125 Messina, Italy; Pediatric Endocrinology Unit, Department of Translational Medical Sciences, University “Federico II” of Naples, Naples, Italy; Department of Pediatrics, University of Turin, Turin, Italy; Department of Pediatrics, University of Modena and Reggio Emilia, Modena, Italy; Department of Paediatrics, Regional Hospital, Bolzano, Italy

**Keywords:** Conversion process, Shifting process, Thyroid autoimmune disease

## Abstract

**Background:**

It is known that Hashimoto’s thyroiditis (HT) may progress to Graves’ disease (GD) and that this phenomenon may be more frequent in the patients with Down syndrome (DS).

**Aims:**

To shed light on the relationships between Down syndrome (DS) and metamorphic thyroid autoimmunity.

**Patients and Methods:**

We reconstructed the conversion process from HT to GD in 12 DS children. All the data recorded at HT diagnosis and throughout the time interval from entry to GD presentation were retrospectively taken from patients’ files, as well as those recorded at GD diagnosis and during the subsequent evolution.

From GD diagnosis all patients underwent methimazole treatment, at a dose that was adjusted on the basis of clinical findings and thyroid tests.

**Results:**

Time interval between HT and GD was not different in the seven patients who received during that time a L-thyroxine (L-T4) treatment than in those who were not treated. After methimazole onset all patients exhibited a prolonged remission of hyperthyroidism. In 8/12 patients this treatment is still being continued 2–7 years after its initiation. The mean methimazole dosage needed to maintain euthyroidism in these eight patients was 0.12 ± 0.02 mg/kg/day.

In the remaining four patients methimazole was withdrawn from 1.9 to 7 years after its initiation and no relapses were recorded 2.0–2.1 years after its withdrawal. These patients developed, 0.1–0.3 years after methimazole withdrawal, a picture of overt hypothyroidism and needed treatment with L-T4, that is now being continued.

No patients needed non-pharmacological therapies.

**Conclusions:**

1) DS children might be incline to manifest over time a phenotypic metamorphosis from HT to GD and to subsequently fluctuate from hyperthyroidism to hypothyroidism; 2) in DS GD may have a mild biochemical and clinical course.

## Background

Hashimoto’s thyroiditis (HT) and Graves’ disease (GD) are autoimmune thyroid disorders (AITDs) which are caused by two distinct and separate paradigms [1] and have been for a long time regarded as two different disease entities. More recent views, in contrast, have considered the hypothesis that these disorders might be two sides of the same coin [2]. In fact, during the last years, it has been sporadically reported that GD and HT may follow one another in the same individuals, due to a sequential phenotypic conversion from GD to HT [2, 3] or viceversa [4–10].

The most common metamorphic scenario regards the evolution from GD into HT, whereas the metamorphosis from HT into GD seems to be less common. This is probably due to the lack, in the individuals with long-standing HT, of a critical mass of functioning thyroid tissue able to react to thyrotropin (TSH) receptor antoantibodies (TRABs) [7].

Among the variables which seem to be able to condition the shifting process from HT into GD, it has been recently reported that the association with either Turner syndrome (TS) or Down syndrome (DS) might play some predisposing role [11]. In fact, when compared with HT children without these chromosomopathies, those with TS- or DS- associated HT were found to be at higher risk of progressing towards GD [11].

In the present study we have retrospectively reconstructed the conversion process from HT to GD and the subsequent evolution of GD in a series of 12 children and adolescents with DS, in order to shed further light on the specific relationships between DS and metamorphic thyroid autoimmunity.

## Patients and methods

### Study population

For the present study, we took into consideration all the children and adolescents with DS who were being followed, during the period 2000–2010, in the pediatric endocrinology centers of our Hospitals owing to a previous diagnosis of HT and developed throughout follow-up a picture of GD.

The criteria for diagnosis of HT were: a) no exophthalmos; b) hypoechogenic thyroid pattern at ultrasonography (US) consistent with AITD; c) positivity for serum thyroglobulin and/or thyroid peroxidase autoantibodies (TgAbs and TPOAbs, respectively) at titers above the upper limits of the reference ranges, as subsequently detailed; d) negativity for TRABs (<1 IU/l).

The criteria for diagnosis of GD were: a) elevated free thyroxine FT4 (>24.4 pmol/l) and suppressed TSH (<0.1 mU/l) serum levels; b) positivity for TRABs (>1.5 IU/l); c) hypoechogenic thyroid pattern at US consistent with AITD; d) no tendency to spontaneous normalization of hyperthyroidism before methimazole treatment.

### Study design

All the data recorded at HT diagnosis and at GD presentation (median time interval 4.5 years, range 0.7–6.5) were retrospectively reconstructed from patients’ files, as well as those recorded during the subsequent evolution from GD presentation onwards.

Since the time of GD diagnosis all the recruited patients underwent a pharmacological treatment with methimazole, at a dose that was periodically adjusted on the basis of clinical findings and thyroid function tests. The median follow-up duration (from HT diagnosis to the last examination) was 8.0 years (range 5.7–10.1).

### Methods

Serum concentrations of TSH (normal range 0.3–4.5 mU/l) and FT4 (normal range 10.3–24.4 pmol/l) were measured by radioimmunoassay methods.

Anti - TPOAbs and anti – TgAbs were measured by electrochemiluminescence immunoassay methods. According to the employed methods values above 20 and 30 IU/ml, respectively, are defined as positive [12]. TRAB serum levels were measured by a second generation radioreceptor assay using the human recombinant TSH receptor. According to the employed method, values below 1.0 IU/l are defined as negative and values above 1.5 IU/l as positive, whilst values between 1.0 and 1.5 IU/l are considered as questionably positive [13].

Thyroid US examinations for assessment of echogenicity were always performed by experienced ultrasonographers with high – resolution machines.

With regard to thyroid function patterns at diagnosis of both HT and GD, they were evaluated according to FT4 and TSH serum levels and classified into the following groups: 1) euthyroidism (both FT4 and TSH within normal limits); 2) overt hypothyroidism (low FT4 together with elevated TSH); 3) subclinical hypothyroidism SH (normal FT4 as opposed to elevated TSH); 4) hyperthyroidism (suppressed TSH, as opposed to elevated FT4).

### Statistical analysis

Results are expressed as mean ± SD or median and range values, as appropriate. Comparisons between groups were performed by Student’s unpaired t test (normally distributed data) or Mann–Whitney U test (non-normally distributed data), as appropriate. Frequency rates were compared by chi-square (χ2) test. Correlations between quantitative variables were assessed using Pearson’s correlation analysis. The level of significance was set at 0.05 for all the statistical assessments.

The study design was approved by the ethical committees of the hospitals participating in our study and the patients or their parents gave informed consent. Appropriate consent was also obtained earlier from the Study Group for thyroid diseases of the Italian Society for Pediatric Endocrinology and Diabetology.

## Results

The DS children and adolescents with the previous diagnosis of HT who developed, during follow-up, a picture of GD were 12 (seven girls). Their ages at the times of HT and GD diagnosis are reported in Table 1.

**Table 1:** Sex, ages, thyrotropin (TSH), free thyroxine (FT4) and thyroid autoantibody serum levels in the 12 patients of our series at diagnosis of both Hashimoto’s thyroiditis (HT) and Graves’ disease (GD)

Patients	Sex	Age (years)	TSH^a^ (mU/l)	FT4^b^ (pmol/l)	TPOAbs^c^ (IU/ml)	TgAbs^d^ (IU/ml)	TRABs^e^ (IU/l)
1	F	HT 3.0	7.10	12.6	95	60	<1.0
		GD 5.5	0.0500	45.1	796	150	23.4
2	F	HT 3.0	19.56	9.9	1310	182	<1.0
		GD 9.0	0.0500	36.8	1300	79	19.0
3	F	HT 3.3	0.07	24.6	57	187	<1.0
		GD 4.0	0.0060	47.8	44	148	85.0
4	M	HT 4.0	8.80	16.8	180	120	<1.0
		GD 9.9	0.0500	66.1	278	199	32.7
5	F	HT 4.0	5.30	12.1	26	28	<1.0
		GD 8.8	0.0001	41.9	149	758	25.2
6	M	HT 5.0	0.05	50.0	284	35	<1.0
		GD 6.1	0.0500	41.6	189	170	22.0
7	F	HT 5.9	5.10	17.4	75	302	<1.0
		GD 11.1	0.0100	31.6	248	55	28.0
8	F	HT 6.0	8.20	10.5	144	104	<1.0
		GD 9.0	0.0500	26.0	39	3	21.0
9	M	HT 8.0	0.01	38.0	278	199	<1.0
		GD 10.0	0.0050	58.7	278	199	32.7
10	M	HT 9.7	9.10	11.0	37	2	<1.0
		GD 13.2	0.0200	29.4	200	200	23.0
11	M	HT 10.0	8.10	10.5	22	119	<1.0
		GD 15.0	0.0010	34.4	45	220	16.0
12	F	HT 13.5	4.30	18.0	23	109	1.0
		GD 20.0	0.0060	25.0	61	117	31.4

^a^normal range 0.3–4.5 ^b^normal range 10.3–24.4

^c^Thyroid peroxidase autoantibodies (normal values <20)

^d^Thyroglobulin autoantibodies (normal values <30)

^e^TSH receptor autoantibodies (normal values <1.0)

The main results are summarized in Table 1.

Only one patient had already entered puberty at HT diagnosis (No.12 of Table 1), whilst all the remaining ones were still prepubertal.

At HT diagnosis only one patient was biochemically euthyroid (No.12), whereas the other ones exhibited different biochemical patterns of thyroid dysfunction: either SH in seven cases (Nos. 1, 4, 5, 7, 8, 10, 11), or overt hypothyroidism (No. 2), or hyperthyroidism in three cases (Nos. 3, 6, 9), All patients showed elevated serum levels of both TPOAbs and TgAbs, except the case No. 10, whose TgAbs were within normal range (Table 1). In accordance with our criteria for diagnosis of HT, TRAB serum levels were initially negative in all cases.

At HT diagnosis five patients with either overt hypothyroidism (No.2) or SH and TSH level higher than 8 mU/l (Nos. 4, 8, 10, 11 of Table 1) underwent a treatment with L-thyroxine (L-T4), that was prolonged for 1.0–6.5 years (median 2.5), whereas no pharmacological therapy was initially started in the remaining seven cases.

In two additional cases, who were initially either euthyroid (No. 12) or hyperthyroid (No. 6), a biochemical picture of overt hypothyroidism became evident only during the time interval between HT diagnosis and GD presentation. This required a L–T4 therapy that was prolonged in both cases for 1.0 year and was withdrawn at the time of GD presentation (Table 1).

A family history of AITD was recorded in only two patients (Nos. 6 and 7). Extra-thyroidal autoimmune diseases were detected in 5/12 children: celiac disease in the cases Nos. 1, 2, 3, 7 and 12; type 1 diabetes in the cases Nos. 1, 3 and 12; juvenile idiopathic arthritis in the patient No. 12.

In the entire cohort time interval between HT diagnosis and GD presentation ranged from 0.7 to 6.5 years (median 4.2). This time interval was not significantly different in the seven patients who had undergone L-T4 treatment than in those who had not been previously treated. In those who had been treated no significant correlation between L-T4 treatment duration and time interval HT – GD was found.

At GD diagnosis hyperthyroid manifestations (tachycardia and/or palpitations, tremor, weight loss, goiter) were observed in 10/12 patients, but only three of them exhibited exophthalmos (Nos. 4, 7 and 9 of Table 1).

TRAB serum concentrations at GD presentation did not significantly correlate with the values of both TPOAbs and TgAbs recorded at the time of HT diagnosis. Furthermore, TRAB serum levels were not significantly different in the seven patients who had been treated with L-T4 during the time interval HT-GD than in those who had not been treated. In those who had been treated no significant correlation between treatment duration and TRAB serum levels was detected.

After the onset of methimazole treatment (at an initial dosage of 0.38 ± 0.12 mg/kg/day, range from 0.25 to 0.53) all the patients exhibited, at varying time intervals, a prolonged clinical and biochemical remission of hyperthyroidism picture. In 8/12 patients (Nos. 2, 4, 5, 7, 8, 9, 11 and 12 of Table 1) this treatment is still being continued 2–7 years after its initiation (median 2.6 years). The methimazole dosage needed to maintain euthyroidism in these eight patients currently ranges from 0.10 to 0.15 mg/kg/day (mean 0.12 ± 0.02).

In the remaining four patients (Nos. 1, 3, 6 and 10) methimazole treatment was withdrawn from 1.9 to 7.0 years after its initiation and no relapses were recorded 2.0–2.1 years after its withdrawal. All these four patients developed, from 0.1 to 0.3 years after methimazole withdrawal, a biochemical picture of overt hypothyroidism and needed replacement treatment with L-T4, that is now being continued since 1.7–2.0 years. These four individuals did not significantly differ from the other ones, either in terms of thyroid function tests and autoantibody serum levels at HT diagnosis or in terms of TRAB serum values at GD presentation.

No patients needed non-pharmacological therapies, such as surgery or radioiodine ablation, during follow-up.

## Discussion

The fluctuations in phenotypic expressions of AITDs, which may sometimes be observed, have been, in recent years, the theme of editorials [14, 15] and Letters to the Editors [4, 5], that raised interesting questions about this peculiar aspect of AITDs. All these comments have regarded a phenomenon that has been hitherto described almost exclusively by means of isolated case- reports [7–9]. To the best of our knowledge, in fact, the study population reported in the present investigation is the second largest patient cohort in which the phenomenon of metamorphic thyroid autoimmunity has been to date described, after the one by Takasu and Mutsushita [10]. Moreover, our study is peculiar in that it has regarded a highly selected population, consisting exclusively of DS young patients.

An evolution from HT to GD is known to be possibly, although infrequently, observed in the natural history of children with HT [6]. Those findings suggested that HT and GD are two ends of the AITD spectrum, from a primarily cell-mediated destruction of thyroid tissue in HT to TRAB – mediated gland activation causing GD [7]. A mechanism, which was guessed to explain the immunological paradigm shift converting HT to GD, is the possible change in the biological activity of TRABs, from predominantly thyroid-blocking antibodies (TBAbs) during the HT phase to thyroid-stimulating antibodies (TSAbs), when GD manifests itself [14]. According to this hypothesis, TSAb emergence after L-T4 therapy might be sufficient to counteract TBAb inhibition [15]. This hypothetic mechanism, however, would not explain why, in the present study, the conversion from HT to GD occurred both in the patients who had been treated with L-T4 and in those who received no treatment. Moreover, it has to be considered that, in our study, TRABs had been sought even at diagnosis of HT and had resulted to be negative in all cases. Therefore, due to these reasons, the hypothesis of a shift in the biological activity of TRABs during the time interval between HT and GD is very unlikely, at least in the patients of our series.

In this study we have found that one third of our patients exhibited, after methimazole withdrawal, a spontaneous change of thyroid function, from hyperthyroidism to hypothyroidism. This finding might be, probably, explained by the hypothesis that the stimulatory effects of TRABs in these cases were overwhelmed by a HT – related severe damage of thyroid tissue [15]. Such hypothesis seems to be particularly relevant to our patients considering that our series consisted exclusively of DS patients, i.e. individuals who are per se more exposed to the risk of thyroid function impairment [16–18].

The relatively high number of children with metamorphic thyroid autoimmunity recruited for this study supports the view that DS children might be particularly prone to manifest over time a phenotypic metamorphosis from HT to GD [11], although the pathophysiological bases of this inclination have not been clarified to date.

Another peculiarity of AITDs in the DS patients of our study population was that clinical course of GD was not very severe, as suggested by both the high remission rate during the first cycle of methimazole treatment and the absence of relapses after methimazole withdrawal. Moreover, the methimazole dosage requested to maintain biochemical euthyroidism was low and alternative non – pharmacological therapies were not needed in any patients of our series. These findings might suggest that in the present cohort of DS patients GD showed a milder clinical and biochemical course, an observation which had been already reported in some previous studies [19, 20].

Finally, in our study population AITDs presented very early, were not associated with familial antecedents of thyroid disorders and showed no gender predominance. All these findings suggest that DS children are per se more exposed to the risk of AITDs, irrespective of age, gender and familial predisposition [19, 21–23].

To sum up, although we are aware that the relatively small study population and the retrospective nature of our study hamper to draw firm conclusions about the significance of our findings, nevertheless we believe that this may be a useful contribution to the specific literature on the metamorphic thyroid autoimmunity, due to the peculiarities of our study cohort.

## Conclusions

We conclude that: 1) DS children might be incline to manifest over time a phenotypic metamorphosis from HT to GD and to subsequently fluctuate from hyperthyroidism to hypothyroidism; 2) in DS GD may have a mild biochemical and clinical course.

## Abbreviations

HT: Hashimoto’s thyroiditis
GD: Graves’ disease
AITDs: Autoimmune thyroid disorders
TSH: Thyrotropin
TRABs: TSH receptor antibodies
TS: Turner syndrome
DS: Down syndrome
US: Ultrasonography
TgAbs: Thyroblobulin antibodies
TPOAbs: Thyroid peroxidase antibodies
FT4: Free thyroxine
SH: Subclinical hypothyroidism
L-T4: L-thyroxine
TBAbs: Thyroid-blocking antibodies
TSAbs: Thyroid-stimulating antibodies
